# A novel tool for individual haplotype inference using mixed data

**DOI:** 10.1186/1423-0127-16-52

**Published:** 2009-06-02

**Authors:** Chen-Pang Lin, Cathy SJ Fann

**Affiliations:** 1 Institute of Public Health, National Yang-Ming University, Taipei, Taiwan; 2 Institute of Biomedical Sciences, Academia Sinica, Taipei, Taiwan

## Abstract

**Background:**

In many studies, researchers may recruit samples consisting of independent trios and unrelated individuals. However, most of the currently available haplotype inference methods do not cope well with these kinds of mixed data sets.

**Methods:**

We propose a general and simple methodology using a mixture of weighted multinomial (MIXMUL) approach that combines separate haplotype information from unrelated individuals and independent trios for haplotype inference to the individual level.

**Results:**

The new MIXMUL procedure improves over existing methods in that it can accurately estimate haplotype frequencies from mixed data sets and output probable haplotype pairs in optimized reconstruction outcomes for all subjects that have contributed to estimation. Simulation results showed that this new MIXMUL procedure competes well with the EM-based method, i.e. FAMHAP, under a few assumed scenarios.

**Conclusion:**

The results showed that MIXMUL can provide accurate estimates similar to those haplotype frequencies obtained from FAMHAP and output the probable haplotype pairs in the most optimal reconstruction outcome for all subjects that have contributed to estimation. If available data consist of combinations of unrelated individuals and independent trios, the MIXMUL procedure can be used to estimate the haplotype frequencies accurately and output the most likely reconstructed haplotype pairs of each subject in the estimation.

## Background

Since the completion of the International HapMap Project, millions of single nucleotide polymorphisms (SNPs) and haplotype information have been deposited into public databases for studies in the fields of population genetics, evolutionary genetics, and complex disease gene mapping. Several studies have demonstrated that haplotypes can provide more power than single markers in detecting associations [1]. However, haplotype information cannot usually be obtained directly from unphased genotype data. It is possible to determine haplotypes using molecular experimental techniques, but such approaches are still expensive and labor intensive. Therefore, haplotype determination from genotype data by statistical methods is used if the estimation is done accurately.

The population-based case-control design is a commonly used design in genetic association studies, in which unrelated cases and controls are collected and compared with respect to the frequencies of some haplotypes. An advantage of this study design is that the implementation is very convenient, since recruiting unrelated individuals is both time- and cost-effective.

One potential disadvantage for the population-based study is due to population stratification which may make an excess of false-positive results. To avoid a deceptive association confounded by population stratification, the family-based designs using relatives of the cases as controls have been proposed. The trio design is the simplest family design, where both parents of the affected subjects are included as family controls. When genotype data for parents are not available, such as in the study of late onset diseases, the unaffected siblings can be included instead. Recruitment, which is the primary disadvantage of family design, usually requires more resources in terms of time and money [2].

A few studies have drawn attention to the association study using both family-based and population-based controls [3,4]. One motivation for this type of study is the supplementation of case-parent trios with additional unrelated controls, if available, to ensure sufficient power to detect association, since parental controls may be hard to recruit, especially for late-onset diseases.

Several statistical and computational approaches have been developed for the inference of haplotype phase from genotype data of unrelated individuals or independent trios, but most programs cannot deal with family-based and population-based controls at the same time. Becker and Knapp [5] proposed a program FAMHAP, which calculates maximum likelihood estimates of haplotype frequencies from general nuclear families via the EM-algorithm. One feature of this program is the possibility of estimating haplotype frequencies from data sets consisting of a combination of unrelated individuals and nuclear families. Nevertheless, this program cannot output the most likely haplotypes pairs of each subject.

In this work, using a MIXture of weighted MULtinomial (MIXMUL) approach, a new procedure based on PHASE [6,7] is proposed for dealing with mixed data sets to estimate the haplotype frequencies and to reconstruct the most likely haplotype pairs of each subject contributed into estimation. We evaluated the MIXMUL procedure with respect to the accuracy of haplotype frequency estimation for the combination data sets. We also considered a few factors, including genotyping error and extent of linkage disequilibrium. The new MIXMUL procedure competes well with the likelihood-based method FAMHAP of Becker & Knapp, which is also applicable to mixed data sets. While FAMHAP can only output haplotype frequency estimates, the MIXMUL procedure can further provide a list of the most probable haplotype pairs for every subject in the mixed data sets.

## Methods

### MIXture of weighted MULtinomial (MIXMUL) approach

We assumed Hardy-Weinberg equilibrium for haplotypes in all subjects and throughout this study and considered a sample consisting of *n*_1 _unrelated individuals and *n*_2 _independent trios. For each subject in the mixed sample, we observed *q *SNPs with alleles 1 and 2 in a specific region of the genome, and *Q *possible haplotypes, with *Q *≤ 2^*q*^. Let *H *= *H*_1_, *H*_2_, ⋯, *H*_*Q *_denote the *Q *possible haplotypes, and a vector  was used to describe the unknown haplotype frequencies, with .

Assuming that the haplotypes from the *n*_1 _unrelated individuals followed a multinomial distribution, the multinomial distribution model was defined as



Let *x*_*i *_be the number of times that haplotype *H*_*i *_occurs, and the vector  be the vector of counts for all haplotypes, with  being the total number of haplotypes. Let *α*_*i *_be the mean haplotype frequency for haplotype *i*, and  be the vector of haplotype frequencies.

Because only parents of each trio would contribute to the estimation, the haplotypes from the founders of the *n*_2 _independent trios also followed a multinomial distribution defined as



Where *β*_*j *_is the mean haplotype frequency for haplotype *j*, and  is the vector of haplotype frequencies. Let *y*_*i *_be the number of times haplotype *H*_*i *_occurs within the *n*_2 _trios, and the vector  be the vector of counts for all haplotypes.

With a weight parameter *λ*, we combined these two sets of haplotype frequencies,  and . Thus, the distribution specified by the mixture of weighted multinomial model is



where *z*_*i *_is the total number of times haplotype *H*_*i *_occurs, and the vector  is the vector of counts for all haplotypes. Thus, we obtained *z*_*i *_as the sum of the number of times that haplotype *H*_*i *_occurs in *n*_1 _unrelated individuals and *n*_2 _independent trios i.e. *z*_*i *_= *x*_*i *_+ *y*_*i*_. Clearly, the multinomial with haplotype frequency vector  specified the same distribution [8].

### Simulation study

We examined the performance of the proposed MIXMUL procedure via simulation studies. The simulations were conducted under settings where the combining of the data is suitable. We generated three dense multi-locus genotype data sets using the program SNaP [9] based on the different haplotype frequency distributions provided from three authentic data sets. The first simulated data set was based on the five SNPs within the N-acetyltransferase 2 gene (*NAT2*) described by Xu *et al *[10]. The haplotype frequencies of the five SNPs were over the 850-bp fragment of *NAT2 *sequenced from each of the 81 individuals to resolve the haplotypes for both chromosomes of each individual. The second simulated data set was the eight SNP haplotype frequencies within the gene *ARHGDIB *on chromosome 12, which were identified from 44 unrelated individuals [11]. The third haplotype frequency distribution was based on the ten SNPs from the original Oxford ACE data described by Zhang *et al *[12]. The ACE data set contained genotypes of 666 individuals with ten SNPs in strong linkage disequilibrium (LD), spanning a very short region (26 kb) within the gene *ACE*.

First, we used PHASE to obtain the count of each haplotype in the "best" reconstruction for the unrelated individuals, i.e. . Then we could obtain an estimated haplotype frequency vector  by . Separately, we used PHASE to obtain the count of each haplotype for the independent trios to form the vector of counts for all haplotypes in these trio families, . A vector of estimated haplotype frequencies  can be obtained by . We then used our proposed MIXMUL procedure to calculate the estimated mixture haplotype frequency vector  as



The weight parameter *λ *was estimated by the maximum likelihood method to obtain the estimator . Given a weight parameter estimate of , a set of estimated haplotype frequencies were obtained by MIXMUL. Based on the set of estimated frequencies for the mixed data set, the MIXMUL procedure can further output the best reconstructed haplotype pairs of each subject contributed into estimation.

### Comparing MIXMUL with FAMHAP

The MIXMUL procedure was developed to deal with mixed data sets consisting of unrelated individuals and independent trios for haplotype frequency estimation and haplotype inference. Using the MIXMUL approach, the weight parameter  was obtained by maximizing the likelihood function of a mixture of two independent multinomial distributions in order to perform haplotype frequency estimation for the mixed data sets. We examined the accuracy for haplotype frequency estimation of the proposed MIXMUL procedure and compared the performance with that of the EM-based FAMHAP proposed by Becker & Knapp, which can also estimate haplotype frequencies for mixed data sets. We set the mixed data sets consisting of 20 unrelated individuals and 5, 10, 15, 20, 25, or 30 independent trios, respectively. Because only parents of each trio would contribute to the estimation, these combinations correspond to a total number of subjects in estimation of 30, 40, 50, 60, 70, and 80, respectively.

To evaluate the performance of MIXMUL and FAMHAP in the presence of genotyping error, we used the ARHGDIB data to generate the simulated data in the existence of genotyping error at the levels of the 0.025, 0.05, and 0.1. The error rates range from 0.025 to 0.1 as suggested by Tintle et al. [13] and Cheng and Lin [14]. To assess the performance of MIXMUL with weak LD extent, we simulated a data set based on eight SNPs with 15 equally frequent haplotypes and average *D' *= 0.3782 for comparing with FAMHAP. On the other hand, we used the ARHGDIB data (average *D' *= 0.9051) and simulated 30 unrelated individuals and 10 independent trios, respectively, to evaluate the estimation accuracy of rare haplotype frequencies (haplotype frequency < 10%) contingent on the same number of subjects utilized.

### Measurement of accuracy

To evaluate the quality of haplotype frequency estimation, the indices *I*_*F *_and *I*_*H *_proposed by Excoffier and Slatkin [15] were used. The first was a similarity measure *I*_*F *_which describes how close the estimated haplotype frequencies are to the actual frequencies and is defined as one minus half of the sum of absolute differences between the true and estimated haplotype frequencies, i.e.,



where  is the estimated frequency of the *i*-th haplotype, and *θ*_*i *_is the true haplotype frequency. *I*_*F *_ranges between 0 and 1 (a value of 1 indicates that the actual and estimated frequencies are identical).

Another measurement *I*_*H *_was used to quantify the effectiveness of computational algorithms for haplotype reconstruction. *I*_*H *_compares the number of haplotypes in a sample and the number of haplotypes detected by an algorithm. In a sample with *N *subjects, the minimum frequency of every true haplotype has to be greater than or equal to , which could be used as a lower bound threshold value for determining the existence of a haplotype. Based on this,



where *k*_*true *_is the number of true haplotypes, *k*_*found *_is the number of identified haplotypes with frequencies above the threshold value, and *k*_*missed *_is the number of true haplotypes not identified. The measure *I*_*H *_also varies between 0 (when none of the true haplotypes is identified) and 1 (when the haplotypes identified are exactly the same as the true haplotypes).

## Results

**Figure 1:**
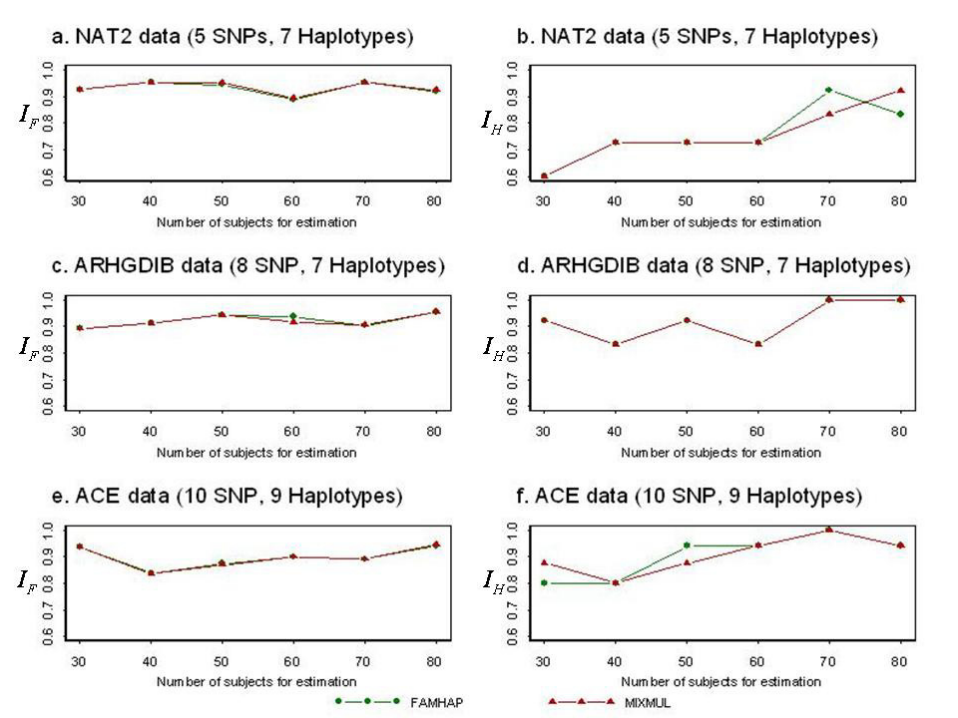
**Performance comparisons of MIXMUL and FAMHAP**. The upper panels (a and b) show the average measures of accuracy based on the NAT2 data. The middle panels (c and d) show the average measures of accuracy based on the ARHGDIB data. The lower panels (e and e) show the average measures of accuracy based on the ACE data. The left panels (a, c and e) and the right panels (b, d and f) show the similarity indices *I*_*G *_and *I*_*H*_, respectively, between the estimated and the actual haplotype frequencies.

The plots in Figure 1 show accuracy comparison of haplotype frequency estimations between MIXMUL and FAMHAP using NAT2 data (panels a and b), ARHGDIB data (panels c and d), and ACE data (panels e and f). Compared to the EM-based program FAMHAP, results were consistent with the notion that MIXMUL has comparable accuracy for haplotype frequency estimation with FAMHAP. Moreover, MIXMUL can output the most likely reconstructed haplotype pairs for every subject into estimation, while FAMHAP only output overall estimated haplotype frequencies.

**Figure 2:**
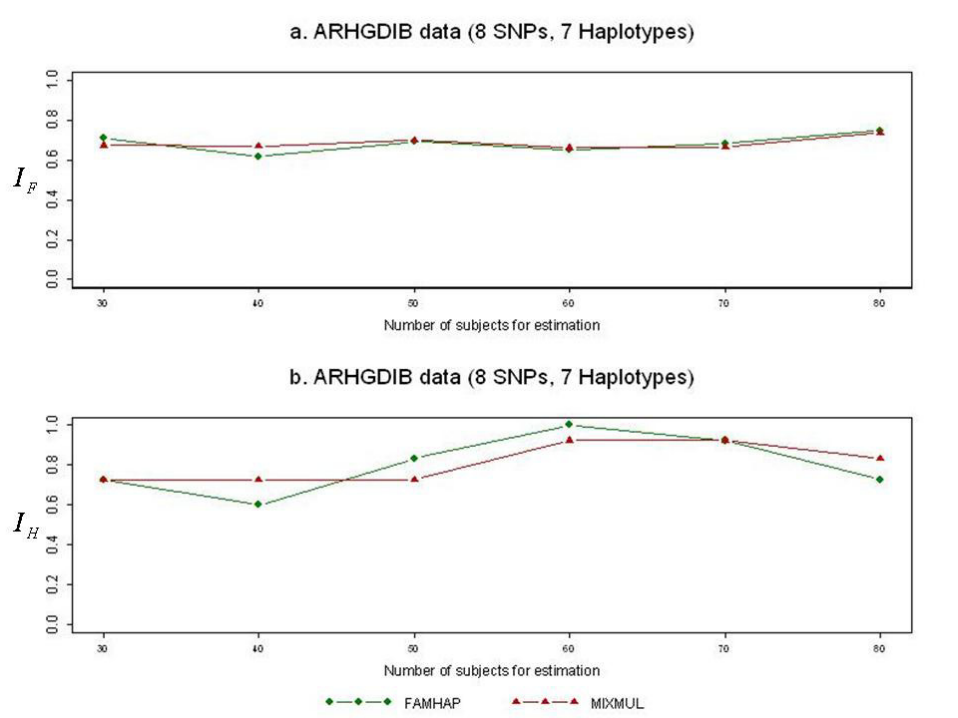
**Performance comparison when genotyping error rate is 0.05**. The graph shows the accuracy indices *I*_*G *_and *I*_*H *_between MIXMUL and FAMHAP based on the ARHGDIB data given the genotyping error rate of 0.05.

It is known that genotyping error can severely affect the performance of haplotype frequency estimation algorithms. Because these results were similar for each level of the genotyping error rate, Figure 2 only shows the results for genotyping error rate of 0.05. They indicated that in most cases, the accuracy using MIXMUL was slightly better than that obtained from using FAMHAP.

**Figure 3:**
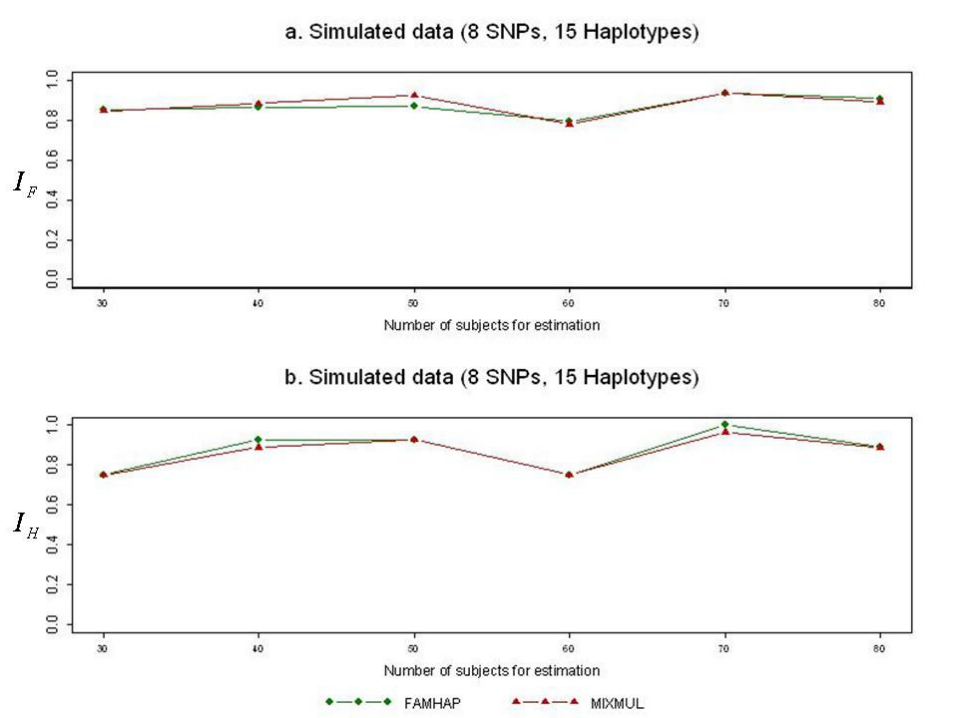
**Performance comparison when LD extent is weak**. The graph shows the accuracy indices *I*_*G *_and *I*_*H *_between MIXMUL and FAMHAP based on the simulated data when the extent of LD is weak (average *D' *= 0.3782).

The extent of linkage disequilibrium (LD) between SNP markers also has an important effect on haplotype-inference accuracy. We evaluated the performance of MIXMUL under a scenario of weak LD extent. Plots of the accuracy measures for the simulated data set were shown in Figure 3. It can be seen that MIXMUL performed well with FAMHAP even when the LD extent was weak.

Most haplotype inference methods can estimate common haplotypes (haplotype frequency > 10%) very accurately when the LD content across the constituent loci is strong, but the performances were lower in estimating rare haplotypes (haplotype frequency <10%). Thus, we used the ARHGDIB data and focused on the estimation accuracy of rare haplotype frequencies contingent on the same number of subjects utilized. Our results showed that the measure *I*_*F *_of 30 unrelated individuals and 10 independent trios were 0.9217 and 0.9720, respectively. Indeed, for rare haplotypes inference, taking family information into account will increase the frequency estimation accuracy.

## Discussion

Haplotype inference for a large number of tightly linked markers through close relatives has drawn much attention in recent years. Several novel methods and programs have been developed. However, it's likely that researchers collected samples consisting of independent trios and unrelated individuals. The MIXMUL procedure proposed here can be conveniently and efficiently utilized to deal with such data sets for haplotype inference. The MIXMUL procedure will also output the most likely reconstructed haplotype pairs of each subject contributed into the estimation.

The current version of the PHASE program (version 2.1) can reconstruct haplotypes from population-based or trio-based genotype data respectively, but it cannot handle mixed data sets consisting of both. On the other hand, the FAMHAP program is able to deal with mixed data sets, however, this program does not provide haplotype inference to the individual level; it only provides overall haplotype frequencies. Based on the outputs of the PHASE program, we proposed the MIXMUL procedure that deals with mixed data sets and infer most probable haplotypes to the individual level by using a weighted function.

Becker and Knapp proposed a program FAMHAP, which calculates maximum likelihood estimates of haplotype frequencies from general nuclear families with an arbitrary number of children via the EM-algorithm. This program can estimate haplotype frequencies from data sets consisting of a combination of unrelated individuals and nuclear families. However, in our simulation, we compared some factors that would affect haplotype frequency estimation, including number of SNP markers, genotyping error rates, and extent of LD. On the basis of our results, the accuracy of haplotype frequency estimation for MIXMUL competes well with FAMHAP when considering all these factors. Furthermore, MIXMUL can provide not only the accurate haplotype frequency estimates, but also the most likely reconstructed haplotype pairs of each subject.

As suggested by other studies [5,16,17], including family information improved the accuracy of haplotype estimation. We examined whether adding families to unrelated individual data could improve the accuracy of haplotype frequency estimation by using MIXMUL. On the basis of our results, the accuracy of haplotype frequency estimation by MIXMUL showed that taking family information or partial family information into account did improve the accuracy of haplotype frequency estimation. However, the accuracy of using more than 60 unrelated individuals for haplotype inference was almost the same as those from using trio or mixed data.

A systematic approach was not used in this study to address the issues because the true haplotype frequencies (derived from experiments) were more suitable for comparison purposes with inferred haplotype frequencies using *I*_*F *_and *I*_*H *_calculated from MIXMUL and FAMHAP. Thus, we selected three published data sets available online where the true haplotype frequencies were derived from experiments. We carried out simulations based on these known frequencies and the number of SNPs ranged from 5 ~10 with linkage disequilibrium coefficient (*D'*) ranged from 0.8522 ~0.9341. The levels of genotyping error rates were 0.025, 0.05 and 0.1 as suggested by other studies [13,14]. By using this approach, we obtained approximate information about the impact of these factors in assessing accuracy in comparison between MIXMUL and FAMHAP.

Incomplete trios (1 parent, one child) in mixed data sets can be analyzed by MIXMUL by setting one parent in a trio as missing. We used the ARHGDIB data to conduct 100 simulations to evaluate the impact of using incomplete trios. The results showed that when using only incomplete trios in the mixed data sets, the accuracy of haplotype frequencies estimation was lower than that obtained from using complete trios (2 parents, one child) because the degree of family information was deficient. For example, given 10 unrelated individuals and 5 incomplete trios, *I*_*F *_was 0.769 and it was 0.825 if these trios were complete. *I*_*H *_was 0.805 for the former and 0.825 for the later. Given 5 unrelated individuals and 40 incomplete trios, *I*_*F *_was 0.925 and it was 0.958 if these trios were complete; *I*_*H *_was 0.928 for the former and 0.985 for the later. The differences of accuracy measurements between using incomplete and complete trios were smaller when the number of subjects used in estimation increased.

## Conclusion

Haplotypes capture LD information in chromosomal regions descended from ancestral chromosomes. Such information is of considerable interest in population genetics and genetic epidemiology studies. With widespread applications of new generations of genotyping techniques, especially high-density SNP arrays, the human genome will eventually be unlocked by linking haplotype information to biomarker and phenotypic data. In current association studies, it is likely that researchers may recruit mixed samples consisting of independent trios and unrelated individuals. However, most existing methods for haplotype inference and frequency estimation cannot cope with these kinds of mixed data sets. Although the EM-based FAMHAP of Becker & Knapp can deal with such kind of data, it cannot reconstruct the haplotype pairs of the individual level. Therefore, in this study we developed the MIXMUL procedure based on the program PHASE to deal with mixed data sets. According to our results, MIXMUL can provide accurate estimates for haplotype frequencies as FAMHAP and further output the probable haplotype pairs in the most optimal reconstruction outcome for every subject that have contributed to estimation. If available data consist of combinations of unrelated individuals and independent trios, the proposed MIXMUL procedure can be used to perform haplotype frequency estimation to obtain accurate haplotype frequency estimates in the mixed sample as well as to output the most likely reconstructed haplotype pairs of each subject into the estimation for further haplotype level association analysis. The MIXMUL procedure is available for download from 

## Competing interests

The authors declare that they have no competing interests.

## Authors' contributions

CL carried out this study and drafted the manuscript. CSJF conceived of the study, and participated in its design and coordination and helped to draft the manuscript. All authors read and approved the final manuscript.
